# Evaluation of Interferon-Gamma Release Assays in the Diagnosis of Recent Tuberculosis Infection in Health Care Workers

**DOI:** 10.1371/journal.pone.0006686

**Published:** 2009-08-24

**Authors:** Irma Casas, Irene Latorre, Maria Esteve, Juan Ruiz-Manzano, Dora Rodriguez, Cristina Prat, Ignasi García-Olivé, Alicia Lacoma, Vicente Ausina, Jose Domínguez

**Affiliations:** 1 Servei de Microbiologia, Hospital Universitari “Germans Trias i Pujol” Fundació Institut d'Investigació en Ciències de la Salut Germans Trias i Pujol, Badalona, Spain; 2 Servei de Medicina Preventiva, Hospital Universitari “Germans Trias i Pujol” Fundació Institut d'Investigació en Ciències de la Salut Germans Trias i Pujol, Badalona, Spain; 3 Servei de Pneumologia, Hospital Universitari “Germans Trias i Pujol” Fundació Institut d'Investigació en Ciències de la Salut Germans Trias i Pujol, Badalona, Spain; 4 Universitat Autònoma de Barcelona, Bellaterra, Spain; 5 CIBER Enfermedades Respiratorias, Badalona, Spain; University of Cape Town, South Africa

## Abstract

**Background:**

Health care workers (HCWs) are a group at risk of latent tuberculosis infection (LTBI). The aims of this study were to determine IFN-γ response by Quanti*FERON*-TB GOLD *In Tube* (QFN-G-IT) and T-SPOT.TB in HCWs, comparing the results with tuberculin skin test (TST); and to analyze the capacity of IFN-γ tests to detect recent *versus* remote LTBI with a prolonged stimulation test (PST).

**Methodology/Principal Findings:**

A total of 147 HCWs were enrolled; 23 of whom were BCG vaccinated. 95 HCWs (64.6%) had a previous positive TST and were not retested; and 52 HCWs had a previous negative TST or were tested for the first time. When we analysed individuals without previous positive TST, the number of positive results for T-SPOT.TB was 12/52 (23.1%); and for QFN-G-IT, 9/52 (17.3%). The global concordance (κ) between T-SPOT.TB and QFN-G-IT with TST was 0.754 and 0.929 respectively. Of individuals with previous positive TST, T-SPOT.TB and QFN-G-IT were negative in 51.6% (49/95) and 62.1% (59/95) respectively, decreasing the concordance to 0.321 and 0.288, respectively. In non-BCG vaccinated HCWs with previous positive TST a positive IFN-γ test was associated with degree of exposure and diameter of TST. PST was performed in 24 HCW with previous positive TST and negative IFN-γ tests. PST was developed in 3 cell cultures stimulated with medium alone, ESAT-6 and CFP-10, respectively. In the third and sixth day of incubation period, part of the supernatants were replaced with complete medium supplemented with (rIL)-2. On day 9, ELISPOT assay was performed. In 14 samples PST was not valid due to not having enough cells. In 8 cases, the response was negative, and in 2 cases positive, suggesting that these patients were infected with *Mycobacterium tuberculosis* in some point in the past.

**Conclusions:**

Both IFN-γ tests showed a similar number of positive results, and concordance between the tests was excellent. None of the tests was affected by prior BCG vaccination. IFN-γ tests are a useful tool for detecting recent infection in HCW population.

## Introduction

Health care workers (HCWs) are one of the groups at risk of *Mycobacterium tuberculosis* infection through occupational exposure [1]. However, the risk varies widely among the various occupational groups and according to their exposure to active tuberculosis (TB) patients. Therefore, the screening of HCWs for latent tuberculosis infection (LTBI) is crucial in an infection control program [2]. Periodical tuberculin skin testing (TST) has been recommended as part of surveillance [3].

However, TST has some known limitations. TST measures cell-mediated immunity in the form of a delayed-type hypersensitivity response to the purified protein derivative (PPD) [4]. However, its specificity is limited due to PPD cross reactivity with the vaccination strain of *Mycobacterium bovis bacilli Calmette-Guérin* (BCG), and several non-tuberculous mycobacteria (NTM) [5]. Therefore, individuals sensitized by previous exposures to NTM or vaccinated with BCG may respond immunologically to PPD.

Tests for *in vitro* diagnosis of LTBI based on the measurement of interferon-gamma (IFN-γ) production from peripheral blood mononuclear cells (PBMCs) and whole blood in response to specific *M. tuberculosis* secreted antigens have been developed. The recent use of the 6-kD *M. tuberculosis* early-secreted antigenic target protein (ESAT-6) and the 10-kD culture filtrate protein (CFP-10) [6] encoded in RD1 (Region of Difference) and TB7.7 [7] encoded in RD11, absent in the BCG strain and in the majority of NTM as stimulating antigens has improved the specificity of the tests.

On the basis of this technology, two commercial IFN-γ tests are essentially available: Quanti*FERON*-TB Gold *In Tube* assay (QFN-G-IT) (Cellestis Limited, Carnegie, Victoria, Australia) and T-SPOT.TB assay (Oxford Immunotec, Abingdon, UK). Both tests have received the final approval from the US Food and Drug Administration (FDA) as a tool for diagnosing *M. tuberculosis* infection. There are some differences between the two tests. QFN-G-IT test is whole blood assay that detects IFN-γ produced by T cells in response to ESAT-6, CFP-10 and TB7.7 using an enzyme-linked immunosorbent assay (ELISA) to measure IFN-γ concentrations in supernatants. In contrast, T-SPOT.TB detects the number of IFN-γ producing T cells after stimulating a definite number of isolated peripheral blood mononuclear cells with ESAT-6 and CFP-10 by means of an enzyme-linked immunospot assay (ELISPOT). In commercially available tests, the whole blood and the isolated T cells are short-stimulated with the specific antigens (16–24 h).

Since the development of the IFN-γ assays, promising results in diagnosing LTBI [8]–[15] and active TB [12], [16], [17] have been published. IFN-γ test results are not affected by BCG vaccination or infection by the most common NTM, and its results have shown to be closer than TST in relation to the degree of exposure to *M. tuberculosis* [14], [18], [19]. In addition, in a large-scale cohort studies recently published [20]–[22], positive IFN-γ assays predicted development of active TB in individuals with recent TB contact. However, limited information is available on the performance of the IFN-γ tests, specially T-SPOT.TB in occupational medicine, when used for screening of HCWs [23]–[28]. No head-to-head comparisons between the two available IFN-γ tests have been performed in HCWs.

On the other hand, the IFN-γ assays are thought to reflect more recent, rather than remote TB infections. This is because activated lymphocytes and effector T cells that produce IFN-γ persist for a limited time in the circulation once the antigen is cleared [29]. It is thought that central memory T cells, but not effector ones, may take several days (rather than hours) to produce effector cytokines [30], [31]. Therefore, contrary to the findings of the TST, in cases of remote infection, the IFN-γ level did not increase during the short period of exposure to the antigen in the ex vivo IFN-γ assay at baseline.

In the present study we investigated the performance of both commercially available IFN-γ tests (QFN-G-IT and T-SPOT.TB) and TST for detecting LTBI in HCWs. Concordance between both test results and association with known risk factors for LTBI were studied. We have also analyzed the capacity of the IFN-γ tests to detect recent *versus* remote TB infection, assessing the effector and memory T cell profiles by means of a prolonged stimulation test.

## Materials and Methods

### Study design and setting

We conducted a cross-sectional study from November 2004 to July 2005 at the Hospital Universitari Germans Trias i Pujol in Badalona, Barcelona, Spain. This is a general hospital with more than 600 beds. Approximately fifty TB patients are treated each year at the hospital and the estimated community incidence of TB is 18.6/100.000 habitants [32].

### Study Population

HCWs were recruited in the course of the routine examinations at the time of the study. HCWs were not enrolled consecutively. Each participant gave written informed consent. The study was approved by the Ethics Committee of the Hospital Universitari Germans Trias i Pujol. Information on the following variables was gathered using a standardised questionnaire: age, gender, reason for testing, degree of occupational exposure to TB (with High being defined as HCWs from wards with≥5 contagious patients per year, HCWs from microbiology laboratory and autopsy wards, and from emergency departments; Medium as HCWs from wards with 2 to 4 contagious patients per year; and Low as HCWs from wards with a maximum of 1 contagious patient per year [33]), BCG vaccination and BCG scar, prior TST (date and result), job category, service and years in the health profession.

### Tuberculin skin test

All new HCWs, who did not have a documented TST result, are tested with the TST during the routine examination at the time of employment in the Preventive Medicine Department [34]. If HCWs had a previous positive TST, we took note of the place and the year, but they were no retested. The Spanish national guideline only recommend repeating TST in HCWs with a previous negative TST [34]. TST was performed by the Mantoux method using 2-TU of PPD RT23 (Statens Serum Institut, Copenhagen, Denmark). Induration was measured 48–72 h after the application. Following Spanish national guideline a diameter equal or greater than 5 mm was considered positive (in BCG vaccinated equal or more than 15 mm) [34]. To reduce the risk of confusion between a booster effect and tuberculin conversion on subsequent testing, individuals with negative initial test results were re-tested within 7 to 10 days and the results of this second test were recorded as the definitive result (two-step tuberculin testing procedure) [35]. TST was administered and read by experienced HCWs. Blood for IFN-γ tests was collected before TST application.

### T-SPOT.TB

Briefly, eight millilitres of blood was drawn from each subject by venopuncture in a vacutainer CPT tube (Beckton Dickinson Diagnostics, Franklin Lakes, NJ). PBMCs were isolated by centrifugation. After centrifugation, PBMCs were washed with GIBCO RPMI 1640 medium (Invitrogen, Auckland, N.Z.) and finally, were re-suspended in GIBCO AIM-V (Invitrogen, Auckland, N.Z.). The test was performed following manufacturer's recommendations. Four wells with a membrane pre-coated with monoclonal antibody to IFN-γ were used for each subject. The assay requires a total of 250,000 viable cells per well. Cells were stimulated in each well with medium alone (as nil control), phytohaemagglutinin (as positive control) and different peptide panels from the specific MTB antigens ESAT-6 (panel A) and CFP-10 (panel B). Plates were incubated for 16–20 hours at 37°C with 5%CO_2_. After the incubation, wells were washed four times with PBS and incubated for 1 hour at 2–8°C with a monoclonal antibody to IFN-γ conjugated with alkaline-phosphatase. After another four washing steps and adding a chromogenic substrate, the presence of reactive antigen specific T cells was revealed as a spot on the well.

Spots were scored by an automated AID ELISPOT plate reader (Lector AID Elispots, Autoimmun Diagnostiks GMBH, Germany). All readings were also manually verified. Subjects were considered positive if there was a positive response to one or both antigen panels. Test wells were scored as positive if they contained at least six spot forming cells (SFC) more than in the nil control well and if this number was at least twice the number of the nil control well. The immunoresponse of each individual was considered adequate if the number of spots in the positive control was 20 or more. The result was interpreted as indeterminate if the number of spots in the positive control well was less than 20 and if the antigen specific wells were negative.

### Quanti*FERON*-TB-GOLD *In Tube*

Briefly, a total of 3 ml of blood was drawn for each patient in three tubes of 1 ml each (nil control, positive control and *M. tuberculosis* specific antigens [ESAT-6, CFP-10 and TB7.7]). Samples were incubated with the stimulating antigens during 16–24 h at 37°C. Afterwards, plasma samples were harvested and the amount of IFN-γ released was measured by an enzyme-linked immunosorbent assay (ELISA), according to the manufacturer's instructions. Raw optical densities were interpreted by using specific software provided by the manufacturer. The result obtained in the nil control was subtracted from the mitogen control and the antigen stimulated samples. The *cut off* value for a positive test was 0.35 IU/mL of IFN-γ in the sample after stimulation with the specific antigens, regardless of the result of the mitogen control. The result of the test was considered indeterminate if an antigen-stimulated sample was negative and if the value of the positive control was less than 0.5 IU/ml after subtraction of the value of the nil control.

### Prolonged T cell stimulation test

The prolonged T cell stimulation test was performed using a protocol previously described and validated to our requirements [36]. The assay was performed with frozen PBMCs, that were thawed and resuspended in 10 ml of RPMI medium (Invitrogen, Auckland, N.Z.). Later, cells were washed and cultured in complete medium (90% RPMI and 10% FBS [PAA Laboratories GmbH, Pasching, Austria] supplemented with penicillin and streptomycin) in wells of 96-well round-bottomed microtiter plates at 37°C with 5% CO_2_. For each patient we prepared three different cell cultures. In the first culture, cells were incubated in the absence of antigen (medium alone); in the second one stimulated with ESAT-6 (Panel A); and in the last one with CFP-10 (Panel B). The final volume of each culture was 0.5 mL. After 3 and 6 day incubation period, 0.25 mL of each culture supernatant was removed and replaced with fresh complete medium supplemented with recombinant human interleukin (rIL)-2 (Roche Diagnostics GmbH, Mannheim, Germany) at a final concentration of 5 U/mL. On day 8, cells were washed one time and cultured again in complete medium without rIL-2. On day 9, 250,000 cells were transferred by well to an ELISPOT plate and stimulated with medium alone (negative control), phytohaemagglutinin (positive control), ESAT-6 (Panel A) and CFP-10 (Panel B) during 16–20 hours at 37°C with 5%CO_2_. On day 10, ELISPOT assay was developed according to manufacturer's instructions.

### Statistical analysis

Concordance between both tests was assessed using the Kappa coefficient. Kappa (κ) values below 0.40 indicate weak correlation, values of 0.41–0.60 indicate good agreement and values above 0.60 indicate strong agreement. The difference in means was detected using Students' t-tests. The difference between number of positives (percentage) among different groups was assessed using Pearson's Chi-square test. Risk factors for a positive test result were defined using an odds ratio (OR). To adjust for multiple variables we used a logistic regression model with IFN-γ tests and TST results as the outcomes. All variables included in the multivariate analysis were determined *a priori* based on an estimation of their significance during the unvariate analysis and biological plausibility. Differences were considered significant when the p value was less than 0.05. All analyses were performed using the SPSS statistical software for windows (SPSS version 15.0; SPSS Inc, Chicago, IL, USA).

## Results

A total of 147 HCWs agreed to take part in the study, and 129 of them (87.1%) were screened with TST at least once in the past. As shown in Table 1, the majority of the participants were women (76.9%) and the median age was 43.3 (range: 22–63 years). Only 23 of the HCWs (15.6%) had received BCG vaccination. The mean duration of years spent in the health care profession was 18.4 (range: 1–43 years), and the exposure was particularly high in 16 cases (10.9%).

**Table 1:** Participants characteristics (n = 147).

	Individuals without previous positive TST^1^ (n = 52)		Individuals with previous positive TST (n = 95)		Total (n = 147)	
	n	%	n	%	n	%
Gender						
Women	40	76.9	73	76.8	113	76.9
Men	12	23.1	22	23.2	34	23.1
Age						
18–29	7	13.5	1	1.1	8	5.5
30–39	29	55.7	14	14.8	43	29.3
40–49	13	25.0	38	40.0	51	34.6
>50	3	5.8	42	44.1	45	30.6
Years in the health care profession						
1–4	7	13.5	1	1.1	8	5.5
5–9	8	15.4	6	6.3	14	9.5
10–14	17	32.7	7	7.4	24	16.3
15–24	16	30.7	44	46.3	60	40.8
>24	4	7.7	37	38.9	41	27.9
BCG vaccination						
No	48	92.3	73	76.8	121	82.3
Yes	4	7.7	19	20.0	23	15.6
Unknown	0	0	3	3.2	3	2.0
Occupational TB^2^ degree exposure						
Low	20	38.5	48	50.5	68	46.3
Medium	24	46.1	39	41.1	63	42.8
High	8	15.4	8	8.4	16	10.9
Job category						
HCW^3^	51	98.1	83	87.4	134	91.2
No HCW	1	1.9	12	12.6	13	8.8
TST results						
Negative	44	84.6	0	0	44	29.9
Positive	8	15.4	95	100.0	103	71.1
Diameter induration TST						
<5 mm	44	84.6	0	0	44	29.9
5–9 mm	5	9.7	16	16.8	21	14.3
10–14 mm	1	1.9	10	10.5	11	7.5
>14 mm	2	3.8	4	4.2	6	4.1
Unknown	0	0	65	68.5	65	44.2
T-SPOT.TB						
Negative	39	75.0	49	51.6	88	59.9
Positive	12	23.1	45	47.3	57	38.8
Indeterminate	1	1.9	1	1.1	2	1.3
QFN-Gold-IT^4^						
Negative	43	82.7	59	62.1	102	69.4
Positive	9	17.3	34	35.8	43	29.3
Indeterminate	0	0	2	2.1	2	1.3

1 Tuberculin skin test;

2 Tuberculosis;

3 Health care Worker;

4 Quanti*FERON*-TB Gold *In Tube*.

95 HCWs (64.6%) had a previous positive TST and were not re-tested. The positive TST result was obtained in 9 cases (9.5%) in the last 5 years, in 10 cases (10.5%) in the last 5 to 9 years, in 14 cases (14.7%) in the last 10 to 14 years, in 59 cases (62.1%) more than 14 years ago, and in 3 cases (3.2) the date was not reported. Of the 52 HCWs with a previous negative TST or who were tested for the first time, 8 (15.4%) resulted TST positive, and 44 (84.6%) were TST negative.

**Figure 1:**
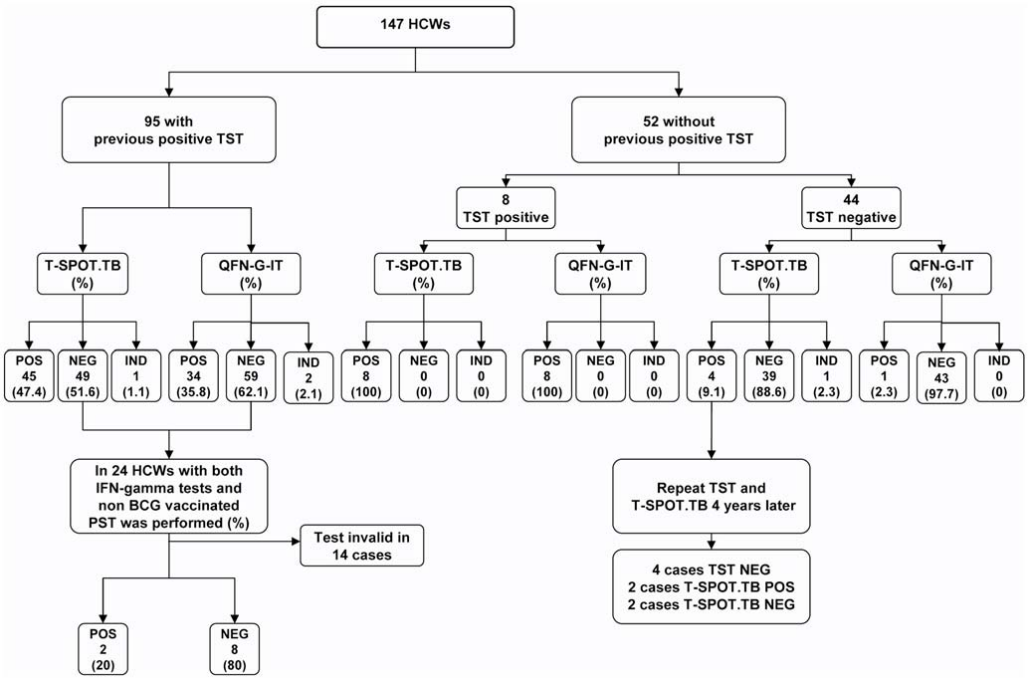
Study flow diagram summarizing study sequence and results. HCW: Health care worker; TST: Tuberculin skin test; QFN-G-IT: Quantiferon-TB Gold *In Tube*; PST: Prolonged stimulation test; POS: Positive; NEG: Negative; IND: Indeterminate.

When we excluded the individuals with previous positive TST, the number of positive results for T-SPOT.TB was 12/52 (23.1%); and for QFN-G-IT, 9/52 (17.3%) (Figure 1). The overall agreement between T-SPOT.TB and TST excluding the patient with indeterminate IFN-γ result was 92.1% (47/51) (κ:0.754; se:0.11) and between QFN-G-IT and TST it was 98% (50/51) (κ:0.929; se:0.07). The agreement between the T.SPOT.TB and the QFN-G-IT was 90.2% (46/51) (κ:0.702; se:0.12). Only 4 HCW were BCG-vaccinated in this subgroup (Table 2).

**Table 2:** Agreement between the tuberculin skin test, T-SPOT.TB and *QuantiFERON*-TB Gold *In Tube* in the different group of patients (excluding indeterminate results).

	TST^1^ vs T-SPOT.TB			TST vs QFN-G-IT^2^			T-SPOT.TB vs QFN-G-IT		
	Agreement (%)	Kappa	SE	Agreement (%)	Kappa	SE	Agreement(%)	Kappa	SE
**All subjects (n = 143)**	90/143 (62.9)	0.321	0.061	84/143 (58.7)	0.288	0.052	123/143 (86.0)	0.692	0.063
BCG vaccination (3 excluded)									
Non BCG vaccinated	76/117 (64.9)	0.349	0.07	74/117 (63.2)	0.345	0.06	101/117 (86.3)	0.701	0.068
BCG vaccinated	11/23 (47.8)	0.169	0.09	8/23 (34.7)	0.085	0.05	20/23 (86.9)	0.650	0.177
**Subjects without previous**									
**positive TST result (n = 51)**	47/51 (92.1)	0.754	0.11	50/51 (98.0)	0.929	0.07	46/51 (90.2)	0.702	0.123
BCG vaccination									
Non BCG vaccinated	43/47 (91.5)	0.749	0.116	46/47 (97.8)	0.928	0.07	41/47 (87.2)	0.695	0.125
BCG vaccinated	4/4 (100)	-	-	4/4 (100)	-	-	4/4 (100)	-	-
**Subjects with previous**									
**positive TST result (n = 92)**	43/92 (46.7)	-	-	34/92 (36.9)	-	-	77/92 (83.7)	0.668	0.077
BCG vaccination (3 excluded)									
Non BCG vaccinated	33/70 (47.1)	-	-	28/70 (40.0)	-	-	59/70 (84.2)	0.682	0.087
BCG vaccinated	7/19 (36.8)	-	-	4/19 (21.0)	-	-	16/19 (84.2)	0.627	0.183

SE: standard error.

1 Tuberculin skin test;

2 Quanti*FERON*-TB Gold *In Tube*.

Among individuals with a previous positive TST, the number of positive results for T-SPOT.TB and QFN-G-IT was 45/95 (47.4%) and 34/95 (35.8%), respectively (Figure 1). Regarding BCG status the number of positive results for T-SPOT.TB in BCG-vaccinated individuals was 7/19 (36.8%) and in non BCG-vaccinated was 33/70 (47.1%). For QFN-G-IT, the number of positive results in BCG-vaccinated was 4/19 (21.1%) and in non BCG-vaccinated population it was 28/70 (40.01%) (Table 2).

In non BCG vaccinated HCWs with a previous positive TST, we evaluated, by both univariate and multivariate analysis (Table 3), the relationship between a positive IFN-γ tests and the risk factors for LTBI. In univariate analysis (P = 0.03; OR: 3.0; 95% Confidence interval [CI]: 1.13–8.15), as well as, in multivariate analysis (P = 0.03; OR: 3.67; 95%CI: 1.07–12.59), only the occupational degree exposure was significant when the outcome was a positive T-SPOT.TB result. For QFN-G-IT, the occupational degree exposure was important but not statistically significant (OR: 2.62; 95%CI: 0.81–8.42).

**Table 3:** Association between tuberculosis risk factors and positive T-SPOT.TB and *QuantiFERON*-TB Gold *In Tube* results in non BCG vaccinated subjects with previous positive tuberculin skin test (n = 70) by means of univariate and multivariate analysis.

Risk factors	T-SPOT.TB		QFN-G-IT^4^		T-SPOT.TB	QFN-G-IT
	Positive n (%)	OR (CI 95%) unadjusted	Positive n (%)	OR (CI 95%) unadjusted	OR (CI 95%) adjusted	OR (CI 95%) adjusted
Gender						
Women	24 (42.1)	1	21 (36.8)	1	1	1
Men	9 (69.2)	3.09 (0.85–11.27)	7 (53.8)	2.0 (0.59–6.75)	0.27 (0.06–1.18)	0.60 (0.16–2.25)
Age years	-	1.01(0.95–1.07)	-	1.03 (0.96–1.09)	1.08 (0.97–1.22)	1.09 (0.98–1.22)
Occupational TB^1^ degree exposure						
Low	13 (34.2)	1	12 (31.6)	1	1	1
High	19 (61.3)	3.0 (1.13–8.15)	15 (48.4)	2.03 (0.76–5.42)	3.67 (1.07–12.59)	2.62 (0.81–8.42)
Years since the previous positive TST^2^	-	1.02 (0.97–1.07)	-	0.99 (0.94–1.05)	1.03 (0.97–1.10)	0.98 (0.92–1.04)
Years in health care profession	-	0.97 (0.92–1.04)	-	1.0 (0.95–1.07)	0.92 (0.82–1.02)	0.96 (0.86–1.06)
Diameter of TST induration						
5–10 mm	4 (33.3)	1	4 (33.3)	1	-	-
>10 mm	5 (50.0)	2.0 (0.35–11.23)	5 (50.0)	2.0 (0.35–11.2)	-	-
Job category						
Non HCW^3^	5 (55.6)	1	3 (33.3)	1	1	1
HCW	28 (45.9)	0.67 (0.16–2.77)	25 (41.0)	1.38 (0.31–6.08)	0.67 (0.10–4.15)	0.51 (0.07–3.56)

CI: Confidence interval; OR: Odds ratio.

1 Tuberculosis;

2 Tuberculin skin test;

3 Health care worker;

4 Quanti*FERON*-TB Gold *In Tube*.

The diameter of TST induration was important in the univariate analysis in both IFN-γ tests, but not significant. The highest diameter of indurations had the greatest percentage of positive IFN-γ tests; however, only 29 of the 95 HCW with previous positive TST had the diameter of induration registered. On the other hand, the results showed no significant association between positive IFN-γ tests and years since the previous positive TST. Nevertheless, the number of responder T cells and the amount of IFN-γ released was higher in the HCWs with previous positive TST results performed in the last 5 years (data not shown).

Of the non BCG-vaccinated HCWs with a previous positive TST and a negative IFN-γ test, a prolonged T cell stimulation test was performed in 24 of them to detect remote infection. In 14 cases the test was not valid because there were not enough cells recovered after the thawing process. For the remaining 10 samples, in 8 cases, although the controls ran well, no response against specific antigen stimulation was obtained after prolonged T cell assay, rendering a negative result. In 2 cases, response against both ESAT-6 and CFP-10 was detected in one case, and only against CFP-10 in the other case. Therefore, the results suggested that in the 20% of cases these individuals were infected in the past.

Concordance between both test results and association with known risk factors for LTBI were also analysed in all the population included in the study. TST was positive in 71.1% (103/147) (Figure 1). Table 4 shows the risk factors associated with a positive TST result. Univariate analysis showed a statistically significant association between positive TST, age and number of years in health care profession (p = 0.001). Interestingly, the non-HCWs showed a higher rate of TST and IFN-γ tests positivities than that shown by HCW. The most likely explanation is that non-HCWs were significantly older than HCWs (P = 0.013), with the mean and SD being 50.4 years (9.5) and 42.6 years (8.7), respectively. There was also association with previous BCG vaccination (OR: 2.37; 95%CI: 0.75–7.46) and gender (OR: 1.80; 95% CI: 0.72–4.55), but the associations were not statistically significant.

**Table 4:** Association between tuberculosis risk factors and positive tuberculin skin test, T-SPOT.TB and *QuantiFERON*-TB Gold *In Tube* results by means of univariate analysis.

Risk factors	TST^1^			T-SPOT.TB			QFN-G-IT^2^			
	Positive n(%)	OR (CI 95%)	P	Positive n (%)	OR (CI 95%)	P	Positive n (%)	OR (CI 95%)	P	
Gender										
Women	74 (67.3)	1	NS	35 (31.8)	1	0,004	29 (26.4)	1	NS	
Men	26 (78.8)	1.80 (0.72–4.55)		20 (60.6)	3.2 (1.47–7.37)		14 (42.4)	2.0 (0.91–4.63)		
Age years	-	1.18(1.11–1.25)	0.0001	-	1.04(1.0–1.08)	0.03	-	1.04(0.99–1.08)	0.05	
Occupational tuberculosis degree exposure										
Low	48 (70.6)	1	NS	20 (29.4)	1	0,04	16 (23.5)	1	NS	
High	52 (69.3)	0.94 (0.46–1.93)		35 (46.7)	2.1 (1.05–4.19)		27 (36.0)	1.82 (0.88–3.80)		
Years in health care profession	-	1.12(1.06–1.18)	0.0001	-	1.0(0.97–1.04)	NS	-	1.0(0.96–1.05)	NS	
BCG vaccination										
No	78 (66.7)	1	NS	45 (38.5)	1	NS	37 (31.6)	1	NS	
Yes	19 (82.6)	2.37 (0.75–7.46)		7 (30.4)	0.7 (0.26–1.83)		4 (17.4)	0.45 (0.14–1.43)		
Job category										
Non HCW^2^	12 (92.3)	1	NS	7 (53.8)	1	NS	4 (30.8)	1	NS	
HCW	88 (67.7)	0.17.(0.02–1.38)		48 (36.9)	0.5 (0.16–1.58)		39 (30.0)	0.97 (0.28–3.32)		

NS: Non significant differences; OR: Odds ratio; CI: Confidence interval.

1 Tuberculin skin test;

2 Quanti*FERON*-TB Gold *In Tube*;

3 Health care worker.

Positive T-SPOT.TB results were obtained in 38.8% of all studied HCW (57/147) in comparison with 29.3% for QFN-G-IT (43/147). T.SPOT.TB was indeterminate in 2 cases and QFN-G-IT was indeterminate in other 2 cases (Figure 1). Table 4 shows the risk factors associated with a positive IFN-γ tests result. On univariate analysis, age, gender and degree of occupational exposure were statistically significant for positive T-SPOT.TB results; in contrast, for QFN-G-IT, only the age was statistically significant. Gender and degree of occupational exposure were important factors but were not statistically significant (OR: 2.0; 95%CI: 0.91–4.63; and OR: 1.82; 95%CI: 0.88–3.80, respectively).

In the multivariate analysis, age showed significant association for positive TST (OR: 1.26; 95%CI: 1.05–12.20) and QFN-G-IT (OR: 1.15; 95%CI: 1.05–1.25) and T-SPOT.TB (OR: 1.14; 95%CI: 1.05–1.24), but occupational TB degree showed significant association only for positive IFN-γ tests (OR: 4.59; 95%CI:1.68–12.51; and OR: 2.72; 95%CI: 1.04–7.13, respectively).

Data on agreement between the TST and IFN-γ test results were available for 143 participants (not including the 4 indeterminate results) (Table 2). The highest number of positive IFN-γ tests was obtained in patients with positive TST over 10 mm, although the differences in the number of positive IFN-γ tests in patients with a TST between 5–9 and over 10 mm were not significant. Regarding the BCG-vaccination status, the overall agreement in the non-vaccinated population was higher than in the BCG-vaccinated population, but the κ values indicate weak agreement in both cases (Table 2). The difference in concordance between TST and QFN-G-IT in non BCG and BCG vaccinated HCWs was significant (P = 0.021); in contrast, between TST and T-SPOT.TB; and between both IFN-γ tests it was not significant (P = 0.189 and P = 0.801, respectively).

## Discussion

Serial TST testing as part of a surveillance of HCWs may induce a boosting phenomenon complicating the TST interpretation. A booster reaction may occur as a result of remote *M. tuberculosis* infection, infection with NTM or prior BCG vaccination [37], [38]. In individuals who undergo serial TSTs, it is possible that, after a negative result in the initial TST, a positive result in the second year of testing may have been the result of a boosted reaction. Although the booster phenomenon is less frequent if the second test is administered more than 2 months after the first TST, it has been described after intervals of 1 year [35], [39] and possible longer [37].

In this respect, IFN-γ tests seem to be a promising alternative to the TST for the diagnosis of LTBI in HCWs. The IFN-γ tests have potential advantages, beyond greater specificity. These include logistical convenience (only require one visit to the healthcare facility, the test result can be available within one day), more objective interpretation of the results, and the ability to perform serial testing without inducing the boosting phenomenon.

When considering HCWs without previous positive TST only, the prevalence of LTBI in this study by IFN-γ tests was higher (T-SPOT.TB: 23.1%, and QFN-G-IT: 17.3%) than by TST (15.4%) with an excellent level of agreement. In univariate analysis, age and number of years in heath care profession were associated with an increased risk for a positive TST. In contrast, for positive IFN-γ tests associations were found with age and the degree of occupational exposure and were not related to previous BCG vaccination. These findings are consistent with previous studies showing that an increased degree of exposure [23], [40], and age [27], [41] were significant risk factors for positive QFN-G tests. Contrary to our results, several studies obtained more positive results by TST than by QFN-G tests, although in the majority of cases the results could be explained by the effect of prior BCG vaccination [23], [24], [26], [27], [41]. Barsegian et al [42] obtained a 1% of positive results by T-SPOT.TB in German radiologist HCWs, and a 34% and 27% of positive TST using>5 mm or>10 mm as a *cut off*, respectively. Authors found that the induration of the TST was significantly higher by foreign births (P<0.001) (all HCWs studied came from areas with a high TB incidence) and previous TST (P = 0.001). Although BCG vaccination did not reach significance, the induration size in vaccinated HCWs increased threefold. In contact studies involving HCWs, TST also obtained a higher number of positive results than QFN-G, however this is attributed to the impact of the BCG vaccination [43], [44]. It has been described that a positive QFN-G result was associated with higher exposure groups [45].

Interestingly, the excellent concordance between T-SPOT.TB and QFN-G-IT with TST (92.1% and 98%, respectively) in patients without previous positive TST, falls dramatically to 46.7% and 36.9%, respectively when compared to those HCWs with previous positive TST. A proportion of negative IFN-γ test cases could be explained by the lack of specificity of TST, with some of the previous positive TST being false-positive results as a consequence of prior BCG vaccination or NTM infection [5]. Indeed, the agreement between TST and IFN-γ test results in non BCG vaccinated HCWs was higher than in BCG vaccinated individuals. Furthermore, we have to consider as potential explanation the fact that we cannot confirm the accuracy of the previous positive TST results performed in other institutions, because the tests were not done under study conditions, and maybe some of them were erroneously considered as positive (the milimetres of induration of the previous positive TST were not recorded). Another limitation is that we have not repeated the TST in the HCWs with a previous positive TST. Therefore, in these individuals we have not compared the results of the IFN-γ with the current TST result. In this respect we asked some HCWs about the possibility of repeating the TST and they all refuse. However, although the capacity to respond positively to tuberculin does no remain constant over the course of an individual's lifetime, and that it can weaken over time; it can never fully disappear. These specific facts could introduce a bias to the study adding false-positive TST results, and thereby increasing discordance.

However, another interesting hypothesis resides in the fact that TST is able to detect both remote and recent TB infection while IFN-γ tests only detect recent infection [28], [46], [47]. This is because after an overnight incubation only activated effector memory T cells that are present in the circulation while the antigen is not cleared have enough time to produce IFN-γ. In contrast, the longer intradermal stimulation of PPD might evoke central memory T cells. Consequently, the IFN-γ released by central memory T cells could be produced and detected by *in vitro* methods after a prolonged stimulation. Therefore, HCWs infected in the past would not respond to *M. tuberculosis* antigen stimulation in a short-incubation period, but might be reactive after a prolonged incubation period [28], [30], [36], [46], [48]. Leyten et al [46] described that among TST positive patients with a history of exposure to *M. tuberculosis*, a 6-day lymphocyte stimulation test was more frequently positive (92% of cases) than the T-SPOT.TB (46%) and QFN-G-IT (33%) with the usual overnight stimulation. In our experience, the 10-day stimulation assay in non-BCG vaccinated HCWs with a previous positive TST and negative IFN-γ tests was positive in the 20% (2/10) of cases, suggesting that these patients were infected with *M. tuberculosis* in some point in the past. Pollock et al [28] reported 19% (7/36) of positive results after an extended stimulation assay in TST-positive HCWs with a negative QFN-G and T-SPOT.TB result. Ferrand RA [48] reported that the 6-day T cell responses to ESAT 6 were greater than responses obtained by ex vivo short-stimulation ELISpot. Recently, Schuck et al [49] exploring new antigens to be candidate biomarkers of LTBI, detected that, in contrast to the short-term single stimulation assay, latency-associated antigens induced IFN-γ expression in memory T cells from the majority of LTBI in the long-term re-stimulation assay.

Although, the hypothesis that short-incubation mainly detects recent or ongoing infection by *M. tuberculosis*, while prolonged-incubation tests seem to be more sensitive for the diagnosis of LTBI has not been totally demonstrated, the published results and our own results make the theory plausible. Indeed, there are findings in accordance with this line of thought from a study of hepatitis C virus showing that short-term ELISPOT responses were not influenced by depletion of memory cells, while the depletion of these memory cells did decrease the antigen-specific responses after prolonged culture [50].

Given that the risk of developing active TB is higher in the first 2 years after infection, the detection of recent TB infection by means of IFN-γ tests in HCWs (mainly immunocompetent) seems to be very useful for targeting the high risk population that really need LTBI.

In our experience, we have detected 5 HCWs with a positive result by IFN-γ test (4 by T-SPOT.TB, and 1 by QFN-G-IT) and a negative TST. These results could be considered as a false-positive IFN-γ result, or as a true LTBI not detected by TST. Indeed, this kind of discrepancy has been previously obtained by other authors. Nienhaus et al [27] in a study that comprised 261 HCWs with exposure to *M. tuberculosis*, 40% of positive QFN-G-IT had negative TST; and Herrmann et al [43], in a contact study involving HCWs, described that, in 2 of 19 cases QFN-G-IT was positive and TST negative. In our study, the patients with negative TSTs and positive IFN-γ tests showed no sign of active TB and were allocated to a 6-month clinical follow-up, without medical therapy. We have continued monitoring the 4 cases with negative TST and positive T-SPOT-TB (the HCW with a negative TST and positive QFN-G-IT stopped working at our institution), and today, 4 years later, all remain healthy, their TST continue to be negative, the T-SPOT-TB is positive in 2 cases, but revert to negative in the other 2 cases. Reversion of QFN-G-IT results [25] has been previously described in a follow-up cohort study of Indian HCWs, although the authors explained that these reversions were related to borderline positive results of QFN-G-IT at the baseline determination. In our study, the two reversions are not associated with a previous borderline T-SPOT-TB result. In one case, the initial response to ESAT-6 and CFP-10 antigens was 4 and 14 SFCs/250,000 cells, respectively, and in the second determination no response was detected. In the other case, the initial response to ESAT 6 antigen was 38 SFCs/250,000 cells, and against the CFP10 antigen was 35. In the second determination after 4 years the no response against the ESAT 6 antigen stimulation was produced, and against CFP-10 antigen only 4 SFCs/250,000 was detected. In addition, spontaneous clearance of TB infection cannot be rejected [51].

The use of IFN-γ tests for serial follow-up of HCWs in order to detect recent infection and avoid the booster effect seems to be an alternative to TST. However, some factors should be taken into consideration: Firstly, it has also been reported that levels of IFN-γ measured by QFN-G-IT remain persistently elevated after treatment for LTBI among HCWs in India [52]. Secondly, Choi et al [53] have described in HCWs QFN-G-IT conversion 2–4 weeks after performing a TST test in positive TST population, but not in the negative ones. Recently, van Zyl-Smit et al [54] have also reported some IFN-γ tests result conversions on day 7 after TST administration. However, they stated that when using a two-step screening strategy it appears safe to develop IFN-γ tests within 3 days of performing the TST. Richeldi et al [55] did not obtain conversion after performing serial TST in negative TST individuals either. Nevertheless, the HCWs who need serial testing will be those with previous negative TST. For serial testing of negative TST HCWs the IFN-γ will be appropiate [25], [53], although further investigation is required.

The main limitation of our study is that we have included a small number of HCWs that allowed the detection of a limited number of significant associations between some risk factors and IFN-γ positivity, especially in patients with a previous positive TST. Nevertheless, despite this limitation, the results obtained are sufficiently consistent to draw conclusions. Our paper not only corroborates the previously published data confirming the use of QFN-G-IT as an *in vitro* test for detecting LTBI in HCWs, but, additionally, we provide new information regarding the use of the T-SPOT.TB and its agreement with the QFN-G-IT and TST highlighting its capacity to detect remote *versus* recent infection.

In summary, both IFN-γ tests showed a similar number of positive results, the concordance between the tests was excellent. In addition, none of the tests were affected by prior BCG vaccination. The decision to select T-SPOT.TB or QFN-G-IT in HCW population will depend essencially on the resources available. The *in vitro* tests required an expert laboratory with trained personnel. The results indicate that the IFN-γ tests are a useful tool for detecting recent infection in HCWs population. The use of IFN-γ tests in the follow-up of negative TST HCWs requires further studies which analyze the meaning of the conversions and reversions results.
